# Immobilization of proteolytic enzymes on replica-molded thiol-ene micropillar reactors via thiol-gold interaction

**DOI:** 10.1007/s00216-019-01674-9

**Published:** 2019-03-21

**Authors:** Sari Tähkä, Jawad Sarfraz, Lauri Urvas, Riccardo Provenzani, Susanne K. Wiedmer, Jouko Peltonen, Ville Jokinen, Tiina Sikanen

**Affiliations:** 1 Drug Research Program, Division of Pharmaceutical Chemistry and Technology, Faculty of Pharmacy, University of Helsinki, P.O. Box 56, Viikinkaari 5E, 00014 Helsinki, Finland; 2 Laboratory of Physical Chemistry, Åbo Akademi University, Porthaninkatu 3-5, 20500 Turku, Finland; 3 Present Address: Fisheries and Aquaculture Research, Nofima - Norwegian Institute of Food, P.O. Box 210, Ås, Norway; 4 Department of Chemistry, University of Helsinki, P.O. Box 55, 00014 Helsinki, Finland; 5 Department of Chemistry and Materials Science, School of Chemical Engineering, Aalto University, P.O. Box 13500, 00076 Espoo, Finland

**Keywords:** Thiol-enes, Microreactors, Microfluidics, Enzyme immobilization, Gold nanoparticles, Mass spectrometry

## Abstract

We introduce rapid replica molding of ordered, high-aspect-ratio, thiol-ene micropillar arrays for implementation of microfluidic immobilized enzyme reactors (IMERs). By exploiting the abundance of free surface thiols of off-stoichiometric thiol-ene compositions, we were able to functionalize the native thiol-ene micropillars with gold nanoparticles (GNPs) and these with proteolytic α-chymotrypsin (CHT) via thiol-gold interaction. The micropillar arrays were replicated via PDMS soft lithography, which facilitated thiol-ene curing without the photoinitiators, and thus straightforward bonding and good control over the surface chemistry (number of free surface thiols). The specificity of thiol-gold interaction was demonstrated over allyl-rich thiol-ene surfaces and the robustness of the CHT-IMERs at different flow rates and reaction temperatures using bradykinin hydrolysis as the model reaction. The product conversion rate was shown to increase as a function of decreasing flow rate (increasing residence time) and upon heating of the IMER to physiological temperature. Owing to the effective enzyme immobilization onto the micropillar array by GNPs, no further purification of the reaction solution was required prior to mass spectrometric detection of the bradykinin hydrolysis products and no clogging problems, commonly associated with conventional capillary packings, were observed. The activity of the IMER remained stable for at least 1.5 h (continuous use), suggesting that the developed protocol may provide a robust, new approach to implementation of IMER technology for proteomics research.

**Electronic supplementary material:**

The online version of this article (10.1007/s00216-019-01674-9) contains supplementary material, which is available to authorized users.

**Figure:**
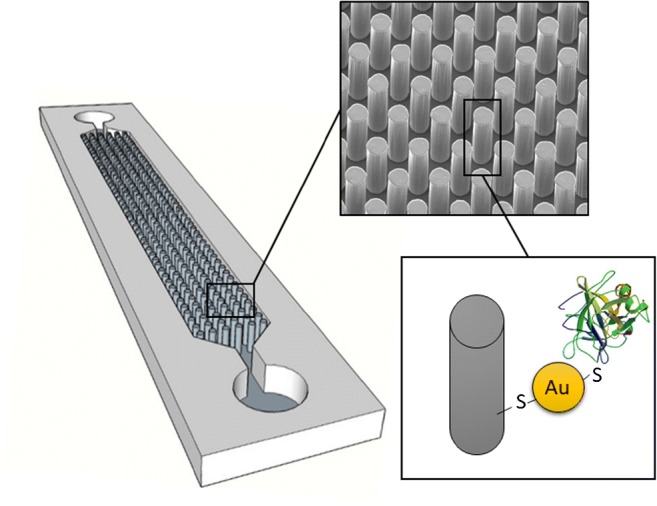
Graphical abstract

## Introduction

Immobilization of proteolytic enzymes (e.g., trypsin, pepsin, and chymotrypsin) on solid support structures packed in capillary channels has gained considerable interest owing to its many benefits over soluble enzyme reactions [1, 2]. Enzyme immobilization on solid support structures omits the need for separation of the enzymes from the reaction solution, which not only simplifies the purification of the reaction products but also allows, for instance, online mass spectrometric (MS) analysis of the proteolytic digest and the reuse of the enzymes, which both result in significant savings in time and costs [3, 4]. Most importantly, enzyme immobilization effectively suppresses autoproteolysis even at high enzyme-to-substrate ratios. In some cases, immobilization also enhances the enzyme stability and activity, which leads to increased conversion rates [5, 6]. However, the enzyme immobilization strategy plays a key role in achieving the high conversion rates with a view to maximizing the amount of bound enzymes and maintaining them active on solid supports [1]. The most common strategies make use of porous polymer monoliths [3, 4] or magnetic beads [6], which are functionalized with covalently bound enzymes and packed in a capillary channel to increase the surface area. Apart from classical esterification, covalent binding may however require harsh conditions that increase the risk of denaturation and loss of enzyme activity. The ester bond, on the other hand, is relatively unstable in aqueous conditions, which may result in leaching of the immobilized enzymes. Alternatively to covalent binding, enzymes have also been physically entrapped inside porous matrixes, but depending on the pore size, these are also prone to enzyme leaching (too large pores) or restricted diffusion of the substrate to the enzyme (small pores) [7]. Any kinds of capillary packing (whether porous matrices or magnetic beads) may also suffer from clogging and reproducibility issues.

Modern microfabrication techniques provide appealing opportunities for the implementation of immobilized enzyme reactors (IMERs). Wafer-scale fabrication processes enable not only parallelism and high degree of system integration, but also customization of the solid support structures with respect to both architecture and the surface chemistry. For instance, dense micropillar arrays have been implemented on silicon in order to increase the surface-to-volume ratio for on-chip chromatographic separations [8–10]. Owing to the possibility to pattern well-ordered micropillar arrays simultaneous to the microchannel network in a reproducible manner, no post-processing (channel packing) is needed and the problems related to clogging can be avoided. Although oxidized silicon readily provides a variety of surface functionalization reactions via silanol groups, which are mostly suitable for covalent coupling reactions, silicon microfabrication as such requires special facilities, including cleanroom instrumentation.

In this work, we introduce rapid replica molding of ordered, high-aspect-ratio micropillar arrays from off-stoichiometric thiol-ene polymers. By mixing the thiol and allyl (“ene”) monomers in off-stoichiometric ratios, the surface chemistry and the mechanical properties of thiol-ene microdevices can be tailored toward a variety of applications [11–13]. In the past, thiol-enes have been used in several applications, including microchip electrophoresis [13, 14] and biosensing [15, 16] devices, protein and DNA arrays [17], and on-chip electrospray ion sources for mass spectrometric detection [18, 19]. The off-stoichiometric thiol-enes (OSTEs) have also been exploited to fabricate porous monoliths inside thiol-ene microchannels followed by covalent coupling of proteolytic enzymes onto both thiol- and allyl-rich surfaces [4, 20]. Here, we make use of the native thiol-ene surface chemistry by preparing thiol-rich micropillar arrays and functionalizing them with gold nanoparticles (GNPs) via the well-characterized and strong thiol-gold interaction [21, 22]. Finally, we demonstrate the immobilization of α-chymotrypsin (CHT) onto GNP-coated thiol-ene micropillars via the enzyme’s free thiol groups (reduced disulfide bridges). The specificity of the thiol-gold interaction between both thiol-rich surface and GNP as well GNP and CHT is also examined, and the robustness of the developed CHT-IMER setup (with respect to flow rate and reaction temperature) is demonstrated by MS analysis of bradykinin hydrolysis products.

## Experimental

### Materials and reagents

Methanol, dimethyl sulfoxide, acetone, tetrahydrofuran, toluene, acetic acid, ammonium acetate, ammonium hydroxide, 5,5′-dithiobis(2-nitrobenzoic acid) (DTNB), phosphate-buffered saline (PBS), α-chymotrypsin from bovine pancreas (CHT, type II, 40 units/mg protein), bradykinin acetate (fragment 1–9), and DL-dithiothreitol were purchased from Sigma-Aldrich (Steinheim, Germany). All reagents and solvents used were HPLC or MS grade (≥ 98.0%). Suspensions of bare GNPs (10 nm, stabilized in 0.1 mM PBS) and dodecanethiol functionalized gold nanoparticles (d-GNPs, 3–6 nm, 0.6–0.9% solid material, 0.01% HAuCl_4_ in toluene) were also from Sigma-Aldrich. Irgacure® TPO-L photoinitiator (2,4,6-trimethylbenzoylphenyl phosphinate) was donated by BASF (Ludwigshafen, Germany). Water was purified with a Milli-Q water purification system (Merck Millipore, Molsheim, France). SU-8 100 negative photoresist (Microchem Corporation, Newton, MA) used for master fabrication was purchased from Micro Resist Technologies GmbH (Darmstadt, Germany). Poly(dimethyl siloxane) (PDMS) used for fabrication of the replication mold was prepared from Sylgard 184 base elastomer and curing agent (Down Corning Corporation, Midland, MI). Trimethylolpropane tris(3-mercaptopropionate) (“trithiol”) (≥ 95.0%), pentaerythritol tetrakis(3-mercaptopropionate) (“tetrathiol”) (≥ 95.0%), and 1,3,5-triallyl-1,3,5-triazine-2,4,6(1*H*,3*H*,5*H*)-trione (“triallyl”) (≥ 98.0%) were used for microchip fabrication and purchased from Sigma-Aldrich (Saint Louis, MO).

### Sample preparation

The activity of immobilized CHT was determined by bradykinin hydrolysis. The stock solution of bradykinin (1 mM in Milli-Q water) was diluted to appropriate concentrations with 20 mM ammonium acetate (pH 8.2) before measurements. The 20 mM ammonium acetate solution used was prepared in deionized Milli-Q water and filtered (0.2 μm) before use in MS analyses. The pH of the ammonium acetate solution was adjusted using 10% (*v*/*v*) ammonium hydroxide. For determination of the specificity of the GNP interactions, phosphate-buffered saline (pH 7.4) was used as the buffer.

### Microchip design and fabrication

**Fig. 1:**
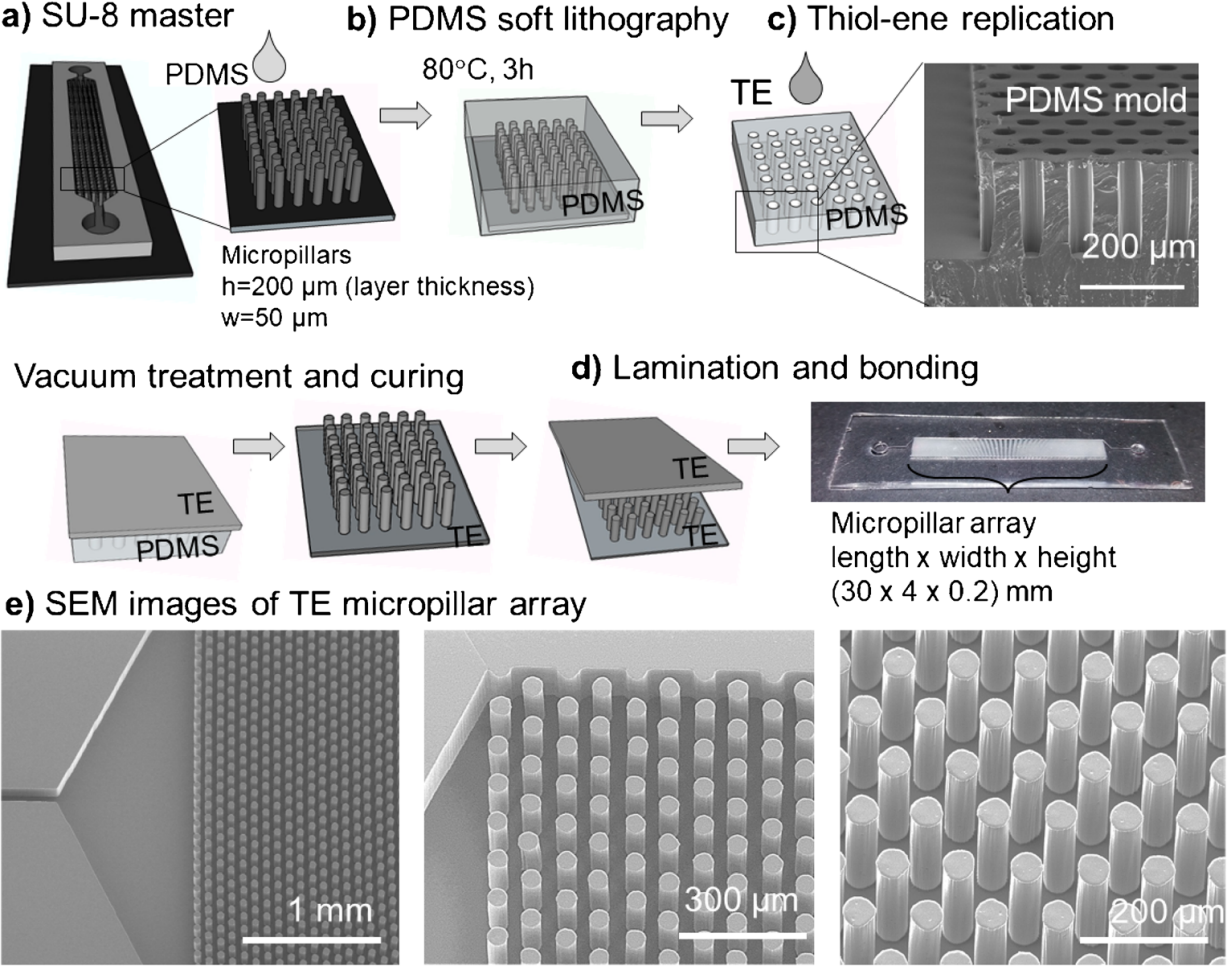
Schematic presentation of the fabrication steps of the thiol-ene micropillar arrays: (a) SU-8 master fabrication in cleanroom, (b) PDMS soft lithography at 80 °C for 3 h, (c) thiol-ene replica-molding: removal of trapped air in vacuum followed by UV curing, and (d) lamination and bonding of the cured thiol-ene layers under UV for 2 min. (e) SEM images of the IMER’s inlet side with triangular opening not containing micropillars (left) with close-up views to the ordered micropillar arrays (middle and right)

The microchip design used in this study comprised of a 30-mm long, 4-mm wide, and 200-μm high microchannel incorporating 14,400 micropillars (average diameter 50.4 ± 0.6 μm, *n* = 10) arranged in a hexagonal geometry. The micropillar array was connected to an inlet and an outlet (both ∅ 2 mm) via tapered, triangular heads not containing micropillars and 3-mm long and 100-μm wide connecting channels (Fig. 1a). The total volume of the IMER was ca. 25 μL. The chip fabrication comprised of four steps: (a) cleanroom fabrication of the SU-8 master, (b) casting of the PDMS mold (soft lithography), (c) replication of the thiol-ene micropillar array under UV light, and (d) bonding of the thiol-ene micropillar array with another thiol-ene (cover) layer (Fig. 1). Apart from the fabrication of the initial SU-8 master (see Electronic Supplementary Material, ESM), all further fabrication steps were carried out in a regular chemistry laboratory (under non-cleanroom conditions). The PDMS molds for thiol-ene replication were prepared by mixing the base elastomer and the curing agent at a ratio of 10:1 (*w*/*w*). After degassing in vacuum for 30 min, the PDMS mixture was cast onto the SU-8 master and cured at 80 °C for 3 h.

The final IMER was fabricated by mixing the “tetrathiol” and “triallyl” monomers in a ratio that yielded 50 mol-% excess of thiol functional groups. This composition was chosen in order to maximize the number of free surface thiols, which is known to increase as a function of increasing excess of the thiol monomer [16]. No photoinitiators were added to the composition to facilitate straightforward bonding of two alike surfaces as described in [12]. However, in the absence of photoinitiators, the curing of thiol-ene compositions with very large excess of the thiol monomer becomes slow and thus the 50 mol-% excess of thiols was the practical upper limit for in this study.

For the specificity tests, also stoichiometric and allyl-rich (50 mol-% excess of allyl functional groups) compositions of “trithiol” and “triallyl” were used. No photoinitiators or other additives were added to any of the compositions. After mixing, the thiol-ene solution was poured onto the PDMS mold, featuring the negative replica of the micropillar array as microwells, and the mold was placed in vacuum for 2–5 min to effectively remove residual air bubbles trapped in the deep microwells (see ESM Fig. S1). Next, the thiol-ene monomer mixture was cured (without cover) under UV light for 5 min by using a Dymax 5000-EC Series UV flood exposure lamp (Dymax Corporation, Torrington, CT, USA, nominal intensity 225 mW/cm^2^). The planar cover layer incorporating only the inlet and outlet holes was prepared in a similar manner. The fully cured cover and bottom (micropillar) layers were then preheated to allow uniform sealing (here, ca. 70 °C was used) and laminated against each other. The bonding was finalized with additional UV exposure through the cover layer for 2 min. The bonding strength was determined by air-pressure tests using an in-house built gas delivery system consisting of an electronic regulator and solenoid valves (SMC Pneumatics Finland Oy, Espoo, Finland). The cured and bonded IMERs were stored at room temperature (RT) in the dark and under atmospheric pressure until use. Characterization of the microstructures was performed by a scanning electron microscope (SEM, FEI Quanta™ FEG, Hillsboro, OR) by attaching the samples onto the sample stage with a carbon-coated double-sided tape and sputtering (Quorum Q150TS, turbomolecular-pumped high-resolution coater, Quorum Technologies, UK) with platinum for 25 s (30 mA) to yield a ca. 5-nm-thick coating.

### Surface functionalization and enzyme immobilization

The immobilization of CHT on the thiol-rich (50 mol-% excess) micropillar array included two steps, both exploiting the thiol-gold interaction. First, the micropillar array was filled with the gold nanoparticle suspension and incubated at 4 °C overnight. Next, the micropillar array was thoroughly rinsed with fresh buffer (20 mM ammonium acetate, pH 8.2) solution followed by another overnight incubation with CHT (1 mg/mL) at 4 °C. Before enzyme incubation, the disulfide bridges of CHT were reduced using 5 mM dithiothreitol (in buffer). Finally, the micropillar-based CHT-IMER was rinsed with 20 mM ammonium acetate (pH 8.2) before determination of the enzyme activity by MS.

### Surface characterizations

#### Titration of free surface thiols

The amount of free thiol groups on micropillar surfaces were quantitated by titration using Ellman’s reagent [23]. Briefly, a concentrated solution of 5,5′-dithiobis(2-nitrobenzoic acid) (DTNB) in PBS buffer was pumped through the micropillar array at a flow rate of 5 μL/min followed by quantitation of the reaction product, 2-nitro-5-thiobenzoate (TNB), by UV absorbance (412 nm) using Varioskan LUX Multimode Microplate Reader (ThermoScientific, Vantaa, Finland). Alternatively, the number of free surface thiols on planar surfaces was determined by submerging thiol-ene slabs (approx. 10 mm × 10 mm × 0.5 mm) into 1 mL of 200 μM DTNB in PBS for 30 min with stirring every 10 min. After 30 min, the thiol-ene slabs were removed and the reaction product TNB was quantitated by UV absorbance as described above. The number of free (reacted) thiols was determined using a molar extinction coefficient for the reaction product of *ε* = 14,150 M^−1^ cm^−1^ [24].

#### Water contact angle

The wetting properties of native thiol-ene and GNP-functionalized surfaces were characterized using a contact angle goniometer (Theta, Biolin Scientific, Espoo, Finland). Advancing and receding water contact angles were determined by the sessile droplet needle method using Young-Laplace fitting. The advancing contact angle was measured by increasing the droplet volume from 2 to 8 μL at a rate of 0.1 μL/s, and the receding angle by decreasing the volume from 8 to 0 μL at a rate of 0.1 μL/s.

#### Atomic force microscopy

An NTEGRA Prima (NT-MDT, Russia) atomic force microscope (AFM) was used for topographical analysis of the native and GNP-functionalized surfaces. The images (1024 × 1024 pixels) were captured in intermittent-contact mode at ambient conditions using gold-coated silicon cantilevers with a nominal tip radius of 10 nm (model: NSG 10, NT-MDT). The scanning rate and damping ratio were 0.2–0.3 Hz and 0.6–0.7, respectively. Image analysis was performed using the SPIPTM image analysis software (Image Metrology).

#### X-ray photoelectron spectroscopy

X-ray photoelectron spectroscopy (XPS) spectra were captured with a PHI Quantum 2000 scanning spectrometer, using monochromatic Al Kα x-ray source (1486.6 eV) excitation and charge neutralization by using electron filament and an electron gun. The photoelectrons were collected at an angle of 45° in relation to the sample surface with a hemispherical analyzer. The average depth of the XPS analysis was in the range of 5–10 nm. The pass energies of 187.85 eV and 29.35 eV were used for collecting survey and high-resolution spectra, respectively. The measurements were done on two different spots for each sample. The atomic concentration (%) of the different elements was derived by calculating the area of the peaks and correcting for the sensitivity factors using a MultiPak v6.1A software (Physical Electronics). The binding energies acquired in the XPS spectra were corrected using the C1s photoelectron peak at 284.8 eV as a reference.

### Determination of enzyme activity

The hydrolysis of bradykinin (Arg-Pro-Pro-Gly-Phe-Ser-Pro-Phe-Arg, MW 1060) on the C-terminal side(s) of phenylalanine(s) was used as the model reaction to monitor the activity of immobilized CHT by electrospray ionization (ESI)-MS. The sample solutions (20 μM bradykinin in 20 mM ammonium acetate, pH 8.2) were infused with a syringe pump at a constant flow rate of 2.5, 5, 10, 15, or 20 μL/min and the reactants were collected for MS analysis in appropriate volumes (typically 100–150 μL per fraction). The effect of reaction temperature (RT vs. physiological temperature 37 °C) was examined by heating the CHT-IMER with a 0.5-Ω resistive heater block (Digi-Key, Thief River Falls, MN) attached to the bottom of the IMER with double-sided tape. The heating temperature was controlled with a DC power supply (Iso-Tech IPS-603, RS Components Ltd., Northants, UK) equipped with a PID temperature controller (type CN743, OMEGA Engineering, Manchester, UK). ESI-MS detection was performed on an Agilent 6330 iontrap mass spectrometer (Agilent Technologies, Santa Clara, CA) using direct infusion (5 μL/min). Before ESI-MS analysis, the collected sample fractions were diluted 1:1 with methanol-water 90:10 containing 0.2% (*v*/*v*) acetic acid. The ion trap was operated in positive ion mode with a capillary voltage set at − 3500 V and end plate offset at − 500 V. Nitrogen produced from compressed air by a Parker nitrogen generator (Cleveland, OH) was used as the drying gas (4.0 L/min, 325 °C). The MS data was acquired over a mass range of *m/z* 100–2000 with a maximum accumulation time of 300 ms using Data Analysis 3.4.

## Results

### Specificity of the functionalization protocol

**Fig. 2:**
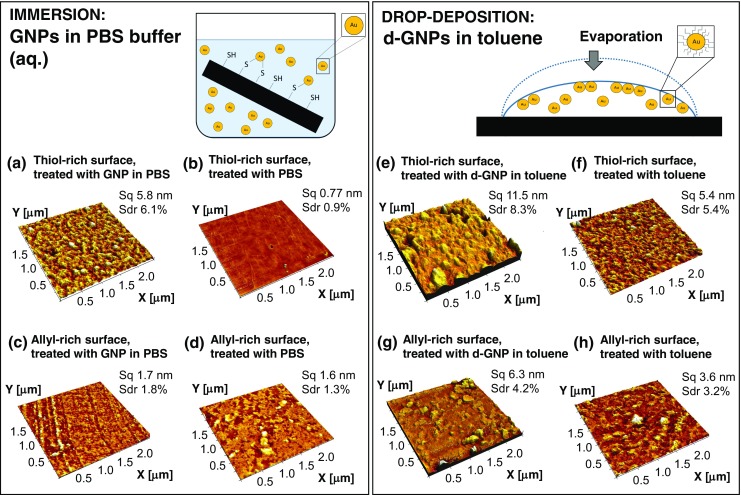
Schematic views of the applied GNP deposition methods, immersion (left) and drop deposition (right), together with AFM images (2.5 μm × 2.5 μm) of the differently treated thiol-ene surfaces: thiol-rich surfaces treated with (a) 10 nm GNP particles in PBS or (b) just PBS, and allyl-rich surfaces treated with (c) 10 nm GNP particles in PBS, or (d) just PBS. Thiol-rich surfaces treated with (e) 3–6 nm dodecanethiol d-GNP particles in toluene or (f) just toluene, and allyl-rich surfaces treated with (g) 3–6 nm dodecanethiol d-GNP particles or (h) just toluene. Sq = root mean square roughness, Sdr = surface area ratio which expresses in percent how much larger the interfacial (real) surface area is compared with the area of the projected (flat) x,y plane

In this work, we examined the possibilities of exploiting the inherent high thiol density of the crosslinked off-stoichiometric (thiol-rich) thiol-enes for efficient functionalization of the micropillar arrays with GNPs. The efficiency of GNP adhesion to native thiol-rich surfaces was first examined by preparing planar GNP-functionalized substrates in two different ways, i.e., via immersion and drop deposition (control method), as illustrated in Fig. 2. The drop deposition method has been shown to result in self-assembly of GNP monolayers upon controlled evaporation (dewetting) achieved by the addition of nonvolatile dodecanethiol ligand [25]. In the drop of the solvent (toluene), the dodecanethiol gold nanoparticles (d-GNPs) form a close-packed monolayer film of floating nanoparticles, which deposits onto the underlying surface upon solvent evaporation. As such, the evaporation approach is not feasible for deposition of GNPs onto enclosed 3D compartments, such as the micropillar arrays, and incubating the pillar arrays with d-GNPs would only result in a nonuniform GNP layer due to nonspecific adsorption of the dodecanethiol on polymer surfaces. Nevertheless, drop deposition was a robust method for preparing GNP monolayers on planar surfaces and thus, a good point of comparison for the preliminary surface characterizations. Since the drop deposition method necessitates the use of organic solvents (such as toluene), we first carried out a solvent compatibility test with two different thiol-rich compositions prepared from either a trithiol or tetrathiol monomer and a triallyl monomer. As a result, thiol-rich compositions prepared using an excess (+ 50 mol-%) of the tetrathiol monomer showed somewhat better stability toward selected organic solvents, particularly toluene, than thiol-rich compositions prepared using the same molar excess of the trithiol monomer (see ESM Table S1). The better solvent tolerance of the tetrathiol-rich compositions was associated with their greater crosslinking degree, which is manifested, e.g., as a decreased oxygen permeability [26], over trithiol-rich compositions. Here, the greater crosslinking degree of the tetrathiol-rich composition likely prevented solvent penetration into the cured (bulk) thiol-ene network, better than trithiol-rich composition, similar to earlier observations [27, 28]. Therefore, the tetrathiol monomer was selected for fabrication of the thiol-ene substrates used in this study.

After incubation with the GNPs (immersion) or d-GNPs (droplet deposition), the surfaces were carefully rinsed with PBS prior to analysis with AFM, XPS, and contact angle goniometry. In all cases, 50 mol-% excess of either the thiol or allyl functional groups was used in the bulk to achieve thiol- or allyl-rich surfaces, respectively. While thiol-rich surfaces were considered ideal for maximizing the amount of thiol-gold interactions, the allyl-rich surfaces provided a good point of comparison with negligible amount of free surface thiols but otherwise very similar surface properties (e.g., in terms of wetting/hydrophobicity [13]). While the immersion approach better resembles the adhesion mechanism in a microchannel and necessitates the thiol-gold interaction, the toluene drop deposition approach facilitates the deposition of d-GNPs on any surface chemistry. Based on topographical analysis by AFM, the deposition of GNPs using the immersion approach (1-h incubation) provided a significant increase in the surface roughness (Sq) from 0.77 to 5.8 nm on thiol-rich surfaces (Fig. 2a vs. b), while their impact on the surface roughness of allyl-rich surfaces was negligible (Sq = 1.6–1.7 nm, Fig. 2c vs. d). In addition to roughness, the differently treated surfaces were examined in terms of the surface area ratio (Sdr) which expresses in percent how much larger the interfacial (real) surface area is compared with the area of the projected (flat) x,y plane. As illustrated in Fig. 2a–d, the effective surface area (Sdr) was also substantially greater for GNP incubated thiol-rich surfaces (Sdr = 6.1%, Fig. 2a) than for any of the controls (Sdr = 0.9…1.8%, Fig. 2b–d). These results suggest that the surface thiols were in a key role in facilitating GNP adhesion. Instead, the drop deposition resulted in much greater overall surface roughness independent of the surface chemistry, although the change was somewhat larger in case of thiol-rich surfaces. Compared with the aqueous PBS treatment (Fig. 2b and d), toluene treatment alone was shown to increase the surface roughness (Sq) and the effective surface area (Sdr) of both thiol- and allyl-rich surfaces (Fig. 2f and h). However, when d-GNPs were included in the toluene incubation, the surface roughnesses of both thiol- and allyl-rich surfaces were further increased from Sq = 5.4 nm to Sq = 11.5 nm and from Sq = 3.6 nm to 6.4 nm, respectively, compared to those of mere toluene-treated surfaces (Fig. 2e and g). Thus, the AFM data clearly evidenced deposition of d-GNPs and formation of the dodecanethiol layer on both thiol- and allyl-rich surfaces when drop deposition method was used, as expected. Generally, the drop deposition method (Fig. 2e vs. g) resulted in somewhat nonuniformly distributed summits and larger, aggregated objects on the surface, which was likely due to nonspecific adsorption of dodecanethiol onto the polymer surfaces preventing proper self-assembly of d-GNPs. Instead, the GNP deposition by the immersion method clearly favored thiol-rich surface (Fig. 2a vs. c) and resulted in a distinct granular morphology, which laid solid grounds for GNP adhesion onto microchannel surfaces.

**Fig. 3:**
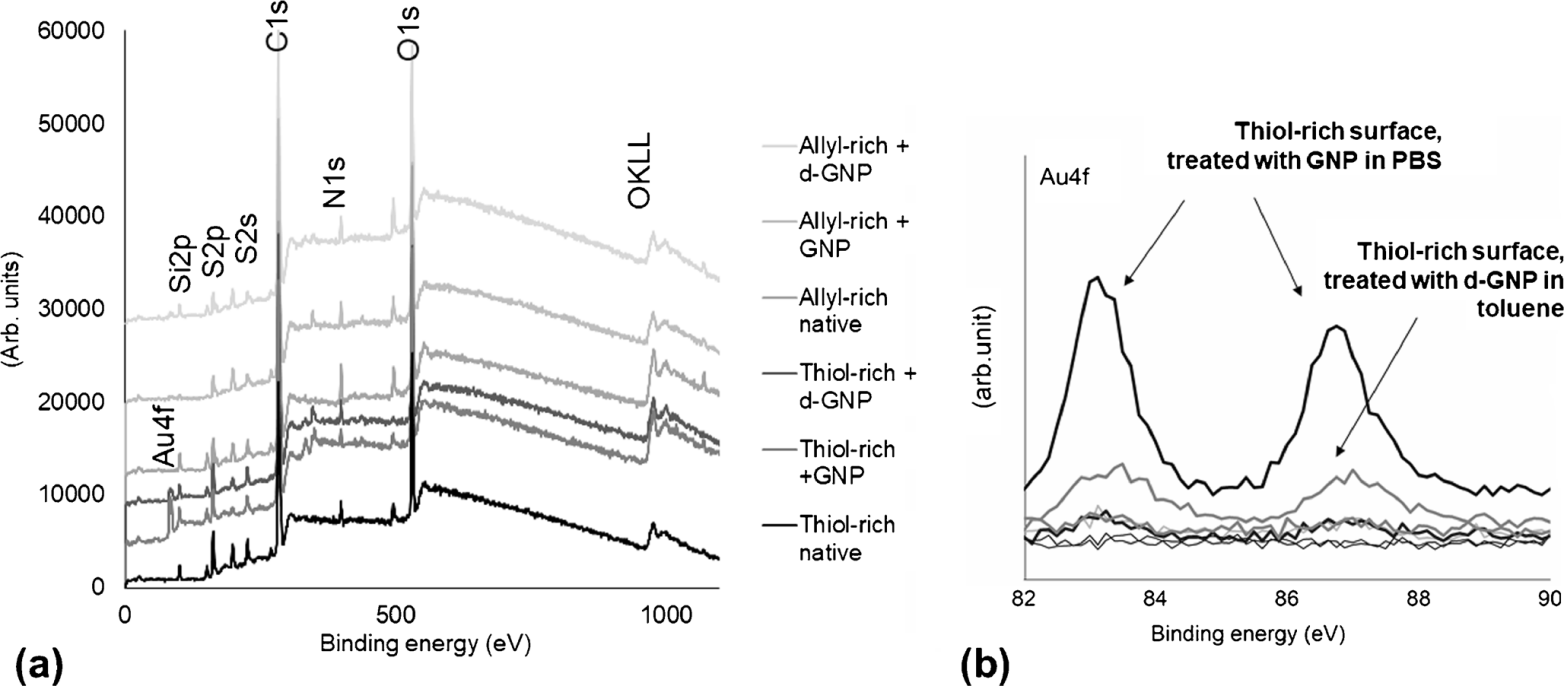
XPS analysis of native and GNP-functionalized thiol-rich and allyl-rich surfaces: (**a**) XPS survey spectra (**b**) High-resolution spectra of Au4f peak

The elemental composition (the presence of gold) of the GNP-functionalized surfaces was further characterized by XPS. The XPS data further confirmed an atomic concentration of ca. 0.6% of gold on thiol-rich surfaces immersed in colloidal GNP suspension in PBS (Fig. 3), while the amount of elemental gold on allyl-rich surfaces was negligible. Surprisingly, the atomic concentration of gold on d-GNP modified surfaces (droplet deposition method) was also significantly small, ca. 0.2% only. Therefore, the d-GNP adhesion on thiol-ene surfaces was further confirmed by water contact angle goniometry. As a result, clear changes in the receding contact angle of both thiol- and allyl-rich surfaces were observed (see ESM Fig. S2), suggesting clear adsorption of d-GNPs based on hydrophobic interactions between the alkanethiol tails and the surface. As also confirmed by AFM, these interactions resulted in very heterogeneous surface topographies and aggregation, which was likely the main reason for the low observed atomic concentration of gold in the XPS experiments. Instead, the GNP deposition via the immersion approach was concluded to provide good grounds for functionalization of through-flow micropillar arrays with a view to enzyme immobilization via thiol-gold interaction. This was further confirmed by contact angle analysis of GNP-treated thiol-rich surfaces following CHT immobilization. A clear change in the surface wettability was observed for CHT-functionalized surfaces with advancing contact angles shifting from the initial values of ca. 80° (native) and 60° (GNP-treated) to clearly below 20°. After CHT incubation, the water droplets formed non-axisymmetric droplets from which exact contact angles could not be measured. This is however typical for hydrophilic and heterogeneous surfaces, such as the CHT-coated surface.

### Fabrication and characterization of the thiol-ene micropillar arrays

Previous studies have shown that the crosslinking degree, and thus, the rigidity and the bonding strength, of thiol-ene polymer networks can be greatly altered by varying both the monomer type (e.g., trithiol vs. tetrathiol) and the (off-)stoichiometric ratio of the thiol and allyl monomers [11, 12, 18]. Upon addition of a photoinitiator to the monomer mixture, the crosslinking occurs faster and typically in a quantitative yield compared to thiol-ene curing without the photoinitiators [29]. As a result, only surfaces with opposite excess of thiols and allyls can be effectively bond to each other [11]. However, by omitting the photoinitiator, also two alike thiol-ene surfaces can be bond together with fairly high bonding strength [12], which indicates that the structure rigidity, for its part, also plays a role. These previous findings further supported the use of the tetrathiol monomer with the triallyl monomer for the fabrication of the micropillar arrays to ensure sufficient rigidity of the replicated, high-aspect ratio (here *h*/*w* ~ 4) micropillar arrays. To maximize the number of free surface thiols toward efficient binding of GNPs, a 50 mol-% excess of thiol functional groups was used in the bulk, which resulted in 162 ± 16 nmol of free thiols per device (*n* = 3 titrations), corresponding to ca. 131 free thiols per nm^2^. The theoretical (calculated) increase in the total surface area and the surface-to-volume ratio, provided by the dense micropillar array incorporating total of 14,400 pillars (each ∅ 50.4 ± 0.6 μm, height 200 μm), was ca. 3-fold and 4-fold, respectively, over a hollow microchannel with identical dimensions (width 3 mm, length 40 mm, height 200 μm).

Consequently, straightforward and good quality sealing of the micropillar arrays with another identical surface was facilitated by omitting the photoinitiator. In this manner, the reverse sides (in contact with PDMS during UV curing) of both layers could be bond together with bonding strengths exceeding 2 bar (the upper limit of our pressure controlled test system). For comparison, micropillar arrays fabricated out of “trithiol” and “triallyl” monomers with equal excess (50 mol-%) of free thiols constantly broke already at 1.5 ± 0.4 bar (*n* = 4 IMERs). The bonding strengths remained unchanged even after delamination (upon heating) and re-bonding of the micropillar arrays, further suggesting that the rigidity of the chosen composition greatly affects the achievable bonding strength.

Before this work, high-aspect thiol-ene micropillar arrays have been achieved via direct photolithographic patterning only [30, 31]. Although the lithography approach allows high feature resolution, it necessitates the use of photoinitiators (and inhibitors), which often complicates adhesive bonding and results in a higher crosslinking degree, and thus lower thiol density on the surface. To examine the effect of the crosslinking degree on the free thiol density, the number of free surface thiols was determined for tetrathiol-rich (50 mol-%) thiol-enes cured (5 min) in the absence and in the presence of the photoinitiator (0.1% TPO-L). As a result, the amount of free thiols dropped dramatically from the initial 190 ± 43 thiols to only 12 ± 1 thiols per nm^2^ (ESM Fig. S3a). Similar decrease in the amount of free surface thiols (15 ± 1 thiols per nm^2^, ESM Fig. S3a) was also achieved by re-exposing the reverse “PDMS side” of the thiol-ene layer for another 5 min (in the absence of the photoinitiator). The impact of curing time was also confirmed by determining the amount of free thiols as a function of UV exposure dose (ESM Fig. S3b). On the average, these thiol densities correspond to ca. 14–18 nmol thiols per device, which is an order of magnitude lower amount than that on a tetrathiol-rich device cured in the absence of the photoinitiator and about an order of magnitude higher amount compared with stoichiometric (2.2 ± 0.3 nmol/device, *n* = 3) and allyl-rich (2.1 ± 0.05 nmol/device, *n* = 2) devices. Since the high density of free surface thiols was found crucial for the efficient coupling of GNPs (as shown in the previous chapter), the replication approach developed herein is likely the only way to achieve sufficiently high coverage of free surface thiols, and thus of GNPs, as it allows fabrication of thiol-ene micropillars without the photoinitiators. The sole critical step in the micropillar replication protocol developed in this study was to remove the air trapped in the deep wells of the PDMS negative mold, as illustrated in ESM Fig. S1, suggesting that the method is robust and feasible for low-cost fabrication of highly ordered micropillar arrays.

### Enzyme immobilization and performance characterization

**Fig. 4:**
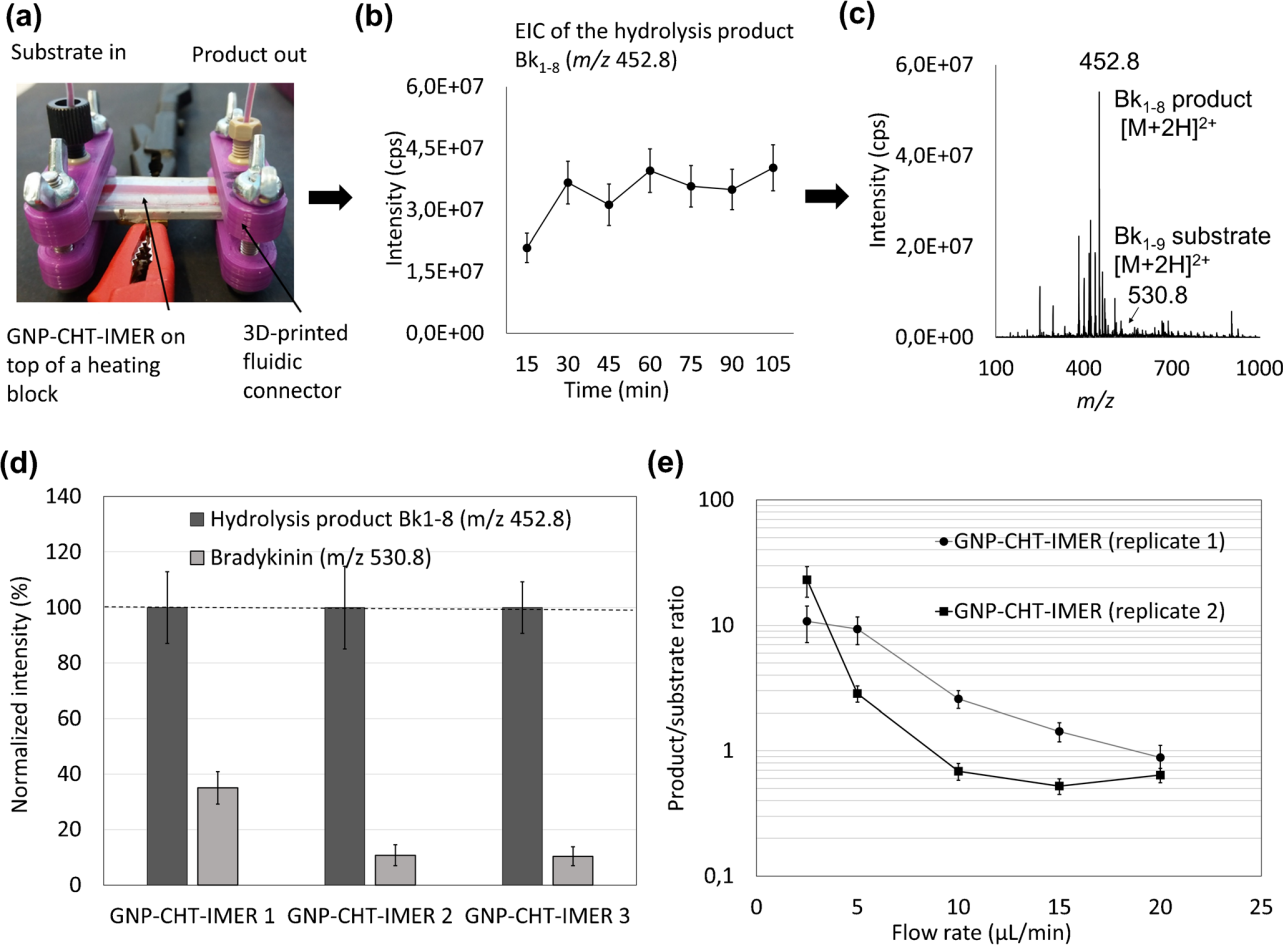
(a) A photograph of the GNP-CHT-IMER in use, assembled with the fluidic couplings, together with (b) the product ion (*m/z* 452.8) stability over time (each fraction 150 μL, flow rate 10 μL/min), and (c) mass spectra obtained at a flow rate of 2.5 μL/min (*n* = 1 IMER). (d) Comparison of the conversion rate (product vs. substrate ion intensity) between three parallel GNP-CHT-IMERs (5 μL/min). (e) Effect of flow rate on conversion rate, i.e., the product/substrate ratio calculated based on the average intensities of *m/z* 452.8 and 530.8 ions from 150 μL fractions collected at different flow rates at RT (*n* = 2 IMERs). The error bars represent the standard deviation of the signal intensity in direct infusion ESI-MS analysis of the particular fraction (single infusion, 1-min period). In all analyses, the substrate solution contained 20 μM bradykinin in 20 mM ammonium acetate (pH 8.2)

The thiol-gold interaction was eventually utilized for functionalization of the thiol-rich (50 mol-% excess) micropillar arrays sequentially with GNPs (to the free surface thiols) and reduced CHT (to the GNP-coated surface via cysteine residues). Previous studies have shown that the size of the nanoparticles has a great impact on the structure and stability of the immobilized enzyme. Although larger nanoparticles (100 nm) may provide better enzyme coverage [32], smaller particles are reported to better preserve the native protein structure [33]. A small particle is also less influenced by gravity effects, which may become relevant when functionalizing 3D topographies, such as the micropillar arrays. Because of these reasons, the 10 nm particles were concluded most feasible for the present study. After functionalization of the micropillar arrays, the activities of the CHT-IMERs were examined by monitoring the intensity ratio of the model substrate, bradykinin (Bk_1–9_, Arg-Pro-Pro-Gly-Phe-Ser-Pro-Phe-Arg), and its main hydrolysis product (Bk_1–8,_ Arg-Pro-Pro-Gly-Phe-Ser-Pro-Phe), which were both detected as double charged ions [M + 2H]^2+^ at *m/z* 530.8 and 452.8, respectively (Fig. 4a–c). Although CHT hydrolyzes bradykinin on the C-terminal side of both phenylalanines, the Phe8-Arg9 bond is the favored cleavage site [34]. The stability of the enzyme activity was shown to be good over a period of at least 100 min (Fig. 4b). After the first fraction (representing the system void volume), the variation of the bradykinin hydrolysis product (*m*/*z* 452.8) intensity between collected fractions was within 9% RSD (*n* = 6 fractions, each 150 μL). The specificity of the enzyme immobilization protocol was examined with the help of two different negative controls, i.e., micropillar arrays functionalized with only GNPs (no CHT) or only CHT (no GNPs), using a flow rate of 5 μL/min (ca. 5-min residence time). As expected, no hydrolysis product was produced in the absence of CHT (ESM Fig. S4a), but some nonspecific binding of CHT was observed in the absence of GNPs resulting in a relatively abundant product peak (Bk_1–8_) (ESM Fig. S4b). However, the catalytic activity of nonspecifically bound CHT (control without GNPs) varied largely across replicates (*n* = 3 IMERs). On the average, the intensity of the product Bk_1–8_ ion (3.4 ± 4.1 × 10^7^ cps, *n* = 3) was just slightly above that of the bradykinin substrate ion (2.8 ± 1.3 × 10^7^ cps, *n* = 3) evidencing insufficient enzymatic conversion. Instead, the more specific CHT immobilization via GNPs was shown to provide substantially higher catalytic activity compared to nonspecifically adsorbed CHT, as expected. On the average, the intensity of the product Bk_1–8_ ion (11.3 ± 3.5 × 10^7^ cps, *n* = 3) was clearly greater than that of the bradykinin substrate ion (2.4 ± 2.3 × 10^7^ cps, *n* = 3). The variation in the catalytic conversion rates (normalized product vs. substrate ion intensities) of the GNP-CHT-IMERs and the CHT-IMERs (negative control) are illustrated in Fig. 4d and ESM Fig. S4c, respectively. The average conversion rates (product/substrate ratio 7.3 vs. 1.1, respectively) were also statistically different (*p* = 0.049) between the different types of IMERs. Next, the impact of the reaction temperature and flow rate (residence time) on the conversion rate (product/substrate ratio) was examined. As expected, increasing the reaction temperature from RT to physiological temperature (37 °C) increased the product conversion rate by about 2-fold (*n* = 2 IMERs) (see ESM Fig. S5), but was typically associated with simultaneous increase in the MS background, which favored the use of RT. However, a greater increase in the product conversion rate was obtained by increasing the residence time (i.e., decreasing the flow rate). Although some variation between IMERs were observed, the same trend was confirmed by two replicate IMERs (Fig. 4e). At the lowest flow rates tested (2.5 μL/min), almost complete bradykinin hydrolysis was observed as illustrated in Fig. 4c. In all, these results evidence that the developed enzyme immobilization strategy combined with the facile microdevice fabrication under non-cleanroom conditions provides a robust approach to implementation of proteolytic IMERs for proteomics research. Owing to the good feature resolution achieved via microfabrication, the well-ordered micropillar arrays provide a convenient approach to microchannel packing by facilitating substantial increase in the surface area for maximum enzyme binding, while avoiding the risk of clogging often associated with porous polymer packing.

## Conclusions

In this work, we introduce rapid replica molding of ordered, high-aspect-ratio, thiol-ene micropillar arrays for implementation of microfluidic immobilized enzyme reactors (IMERs) by exploiting thiol-gold interaction. The replica-molding method developed herein provides a straightforward approach for the fabrication of ordered micropillar arrays in non-cleanroom conditions. The possibilities to avoid the use of photoinitiators and to tune the thiol-ene surface chemistry via off-stoichiometry enable not only straightforward bonding but also good control over the number of free surface thiols available for GNP binding. Owing to the vast excess of thiol functional groups, we were able to bind GNPs on the native thiol-rich micropillars in an efficient manner so that these could be further exploited to immobilizing CHT also based on thiol-gold interaction between the GNPs and the thiol residues of the enzyme. Compared with microchannel packing with porous polymer monoliths or magnetic beads, the well-ordered, microfabricated pillar arrays allowed us to avoid the common pitfalls, such as clogging commonly associated with post-processed microchannel packings. The method qualification evidenced that the developed CHT-IMERs performed proteolytic hydrolysis (of bradykinin) in a robust and stable manner at RT and physiological temperature. The product conversion rate was most dependent on the flow rate (residence time), and almost complete (product/substrate ratio > 10) hydrolysis was achieved at a residence time of as short as 10 min (2.5 μL/min). Furthermore, the activity of the IMER remained stable for at least 1.5 h (continuous use), suggesting negligible leakage of CHT out of the IMER. As the enzymes were firmly immobilized, no further purification of the reaction solution was required prior to mass spectrometric detection. In all, the developed protocol is significantly straightforward, yet robust, while being also somewhat universal, since it can be applied for the immobilization of any proteolytic enzymes by their thiol residues. These are the main advantages of the developed IMER technology, which are likely to provide new opportunities for modern proteomics research.

## Electronic supplementary material

ESM 1 (PDF 492 kb)
