# Supplementary material for: Immobilization of proteolytic enzymes on replica-molded thiol-ene micropillar reactors via thiol-gold interaction
Source: Anal Bioanal Chem. 2019 Mar 21;411(11):2339–49. doi: 10.1007/s00216-019-01674-9 (PMC6459972; doi:10.1007/s00216-019-01674-9)
Supplement: Supplementary file 1 — (PDF 492 kb) [file 216_2019_1674_MOESM1_ESM.pdf]

Analytical and Bioanalytical Chemistry

Electronic Supplementary Material

Immobilization of proteolytic enzymes on replica-molded thiol-ene micropillar reactors via thiol-gold interaction

Sari Tähkä, Jawad Sarfraz, Lauri Urvas, Riccardo Provenzani, Susanne K. Wiedmer,
Jouko Peltonen , Ville Jokinen, Tiina Sikanen

Fabrication of SU-8 masters

Silicon wafers used for master fabrication were first dipped into hydrofluoric acid to remove any native oxide and dehydrated overnight at 120°C. SU-8 100 (Micro Resist Technology, Germany) was spin coated (1500 rpm, 30 s) and soft baked on a hotplate (65°C for 25 min followed by 95°C for 3.5 h) to yield a 200-µm-thick SU-8 layer. The layer was then UV exposed with a dose of 1.35 J/cm^2 on the MA-6 mask aligner (Süss Microtec, Garching, Germany). Post exposure bake was done on the hotplate (65°C for 1 h followed by a slow 4-hour ramp back to room temperature). The slow ramp was used to minimize the thermal stress that can lead to adhesion loss of the thick SU-8 structures. The development was done in propylene glycol methyl ether acetate for 75 min. After development, the master was hard baked on the hotplate at 150°C for 3 h to improve the adhesion. Finally, the master was coated by a nominally 40-nm-thick fluoropolymer layer for anti-adhesion. The fluoropolymer was deposited by PECVD (Plasmalab 80+, Oxford Instruments, UK), out of CHF_3 precursor.

Optimization of replica-molding of high aspect ratio thiol-ene micropillars

To ensure good uniformity of the thiol-ene micropillar array (height), a vacuum step was incorporated to the replica-molding protocol prior to thiol-ene curing under UV. The purpose of the vacuum treatment was to suck the air entrapped in the deep PDMS microwells (negative replicas of the micropillars) prior to UV curing. The effect of vacuum treatment is illustrated in Fig S-1. Due to the vacuum treatment, all PDMS microwells were completely filled with thiol-ene, which resulted in a uniform micropillar height over the entire array. The micropillar diameter was also well replicated from the initial SU-8 master (49.9±0.8 µm, n=10) onto PDMS (50.5±0.7 µm, n=10) and thiol-ene (50.4±0.6 µm, n=10).

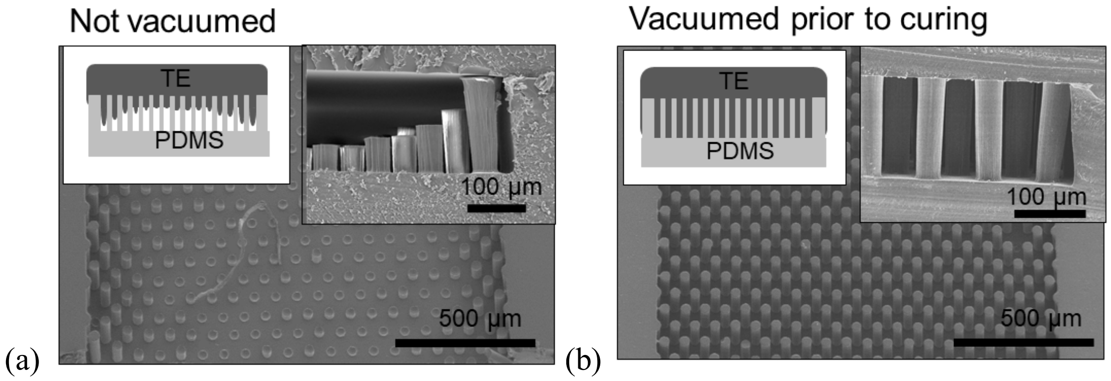

Fig. S1 The effect of vacuum treatment on the uniformity of the micropillar array. SEM images of (a) a micropillar array not kept in vacuum and (b) a micropillar array kept in vacuum for 2-5 min prior to curing

Solvent compatibility

The effects of five different organic solvents on two different thiol-rich (50 mol-% excess) thiol-ene compositions, fabricated from either a trithiol or a tetrathiol monomer and cured for 5 or 10 min, were examined. The solvent compatibilities of the materials were evaluated by visual monitoring of the quality of the cured thiol-ene plates (thickness 0.5 mm, A=1 cm^2) immersed in 1 mL of each solvent for 1 h. If no visual damage was observed with 1h, the solvent exposure was continued for 4 days. The tetrathiol-rich composition was shown to be more stable toward toluene and acetone compared with the trithiol-rich composition, whereas dimethyl sulfoxide, tetrahydrofuran, and dichoromethane caused visible degradation of both compositions.

Table S1 Solvent compatibility of thiol-ene samples fabricated by mixing either trithiol or tetrathiol monomers with triallyl monomers in a ratio yielding 50 mol-% excess of thiol functional groups. √ signifies good resistance and solvent compatibility, ~ limited resistance (cracking) and × poor stability (dissolving/degradation)

Solvent	***Trithiol-rich***	***Tetrathiol-rich***
Dimethyl sulfoxide	x	~
Tetrahydrofuran	x	x
Dichloromethane	x	x
Toluene	~	√
Acetone	x	√

Effect of gold nanoparticle deposition on the wetting properties

The efficiency of gold nanoparticles (GNPs, 10 nm, in phosphate buffer saline) and dodecanethiol-functionalized particles (d-GNPs, 3-6 nm, in toluene) immobilization on thiol-rich thiol-ene plates was examined by advancing and receding water contact angle measurement (Fig S-2) and compared to that of allyl-rich thiol-ene plates. As expected, the d-GNPs were shown to attach on both thiol-rich and allyl-rich thiol-ene surfaces, as evidenced by the clear reduction in the hysteresis (as the result of increased receding contact angle). Instead, binding of GNPs did not significantly affect the contact angles compared with native surfaces and thus, AFM and XPS analyses were required to distinguish between GNP binding on thiol-rich and allyl-rich thiol-ene surfaces.

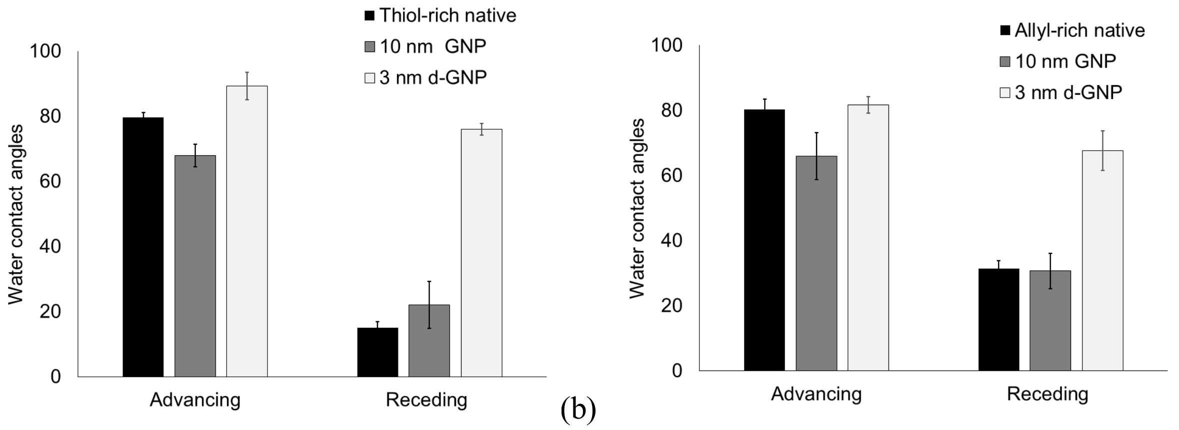

(a) (b)

Fig. S2 Advancing and receding water contact angle analyses of (a) thiol-rich and (b) allyl-rich surfaces. Error bars represent the variation between n=3 experiments

Effect of crosslinking degree on the amount of free surface thiols

The amount of free surface thiols was determined by using the Ellman's reagent [1] as described in the main text. The effect of photoinitiator and an additional UV exposure on the amount of free surface thiols on a thiol-rich (+50 mol-%) surface is illustrated in Figure S3-a, and the effect of curing time on the number of free surface thiols on a stoichiometric surface in Figure S3-b. A clear trend between decreasing amount of free surface thiols and increasing crosslinking degree (as the result of added photoinitiator or increasing UV dose) was observed.

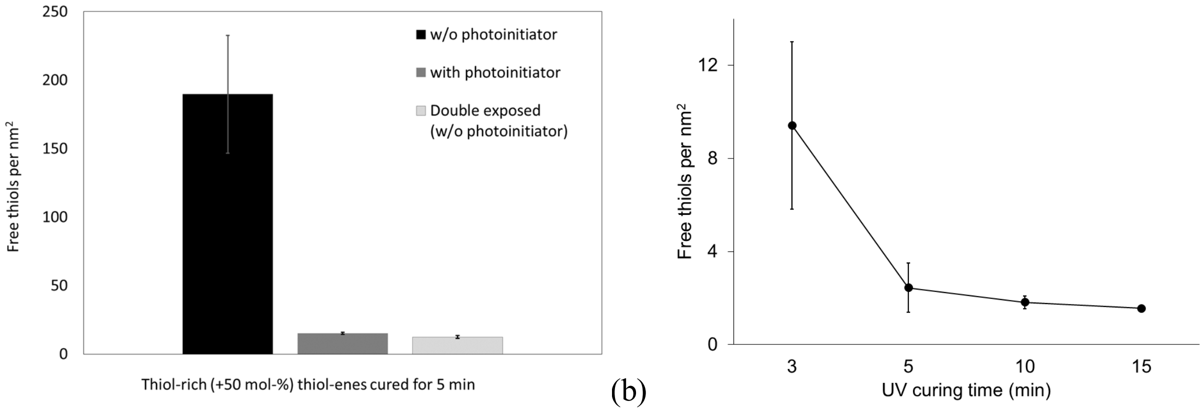

(a) (b)

Fig. S3 (a) Effect of photoinitiator and an additional UV curing (double exposed) on the amount of free surface thiols on a thiol-rich (+50 mol-%) thiol-ene surface (n=3 replicate samples each). The thiol-ene slabs were cured for 5 min with and in the absence of the photoinitiator (0.1 % TPO-L). (b) Effect of UV curing time on the amount of free surface thiols on thiol-ene surfaces prepared using the tetrathiol and triallyl in a stoichiometric ratio (n=3 except for 15 min experiment, which is average value from n=2 experiments)

Specificity of enzyme immobilization

IMERs prepared via incubation with only GNPs (no CHT) or only CHT (no GNPs) were tested for their catalytic efficiency toward bradykinin hydrolysis. As expected, the IMERs lacking the enzyme showed no reaction product (Fig S-4a), whereas IMERs lacking the GNPs contributed to bradykinin hydrolysis suggesting nonspecific adsorption of CHT to the native thiol-ene surface (Fig S-4b).

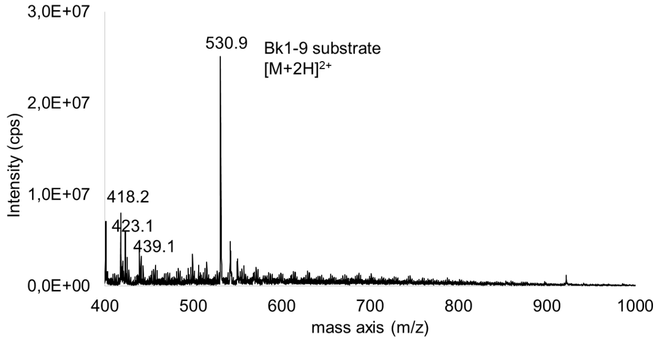

(a)

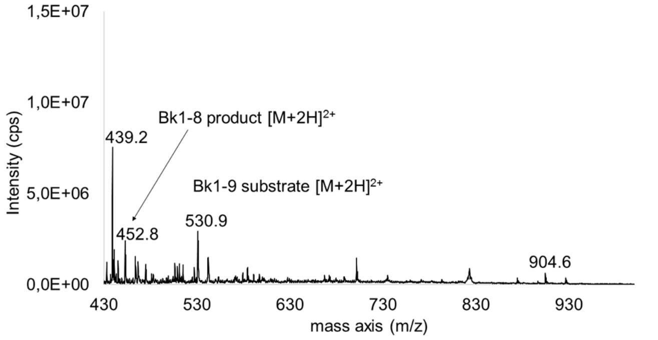

(b)

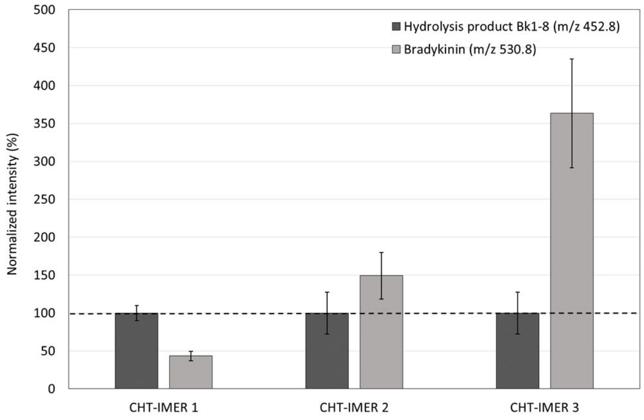

(c)

Fig. S4 ESI-MS analysis of the reaction solutions collected from IMERs incubated with (a) only GNP (no CHT) or (b) only CHT (no GNP). (c) Comparison of the catalytic efficiency of three parallel IMERs (all negative controls) incubated with only CHT (no GNP). The bars represent the average signal intensities of bradykinin and its hydrolysis product, and the error bars the signal variation, over a 1 min period. The flow rate of the substrate solution (20 µM bradykinin in 20 mM ammonium acetate, pH 8.2) was 5 µL/min and all reactions were performed at room temperature

Effect of temperature on the efficiency of bradykinin hydrolysis reaction

The effect of reaction temperature on the efficiency of the CHT-catalyzed bradykinin hydrolysis is illustrated in Figure S-5 indicating ca. 2-fold increase in product/substrate conversion rate from $(2.2\times10^7$ cps) / $(2.0\times10^6$ cps)~10 to $(2.4\times10^7$ cps) / $(1.0\times10^6$ cps)~20.

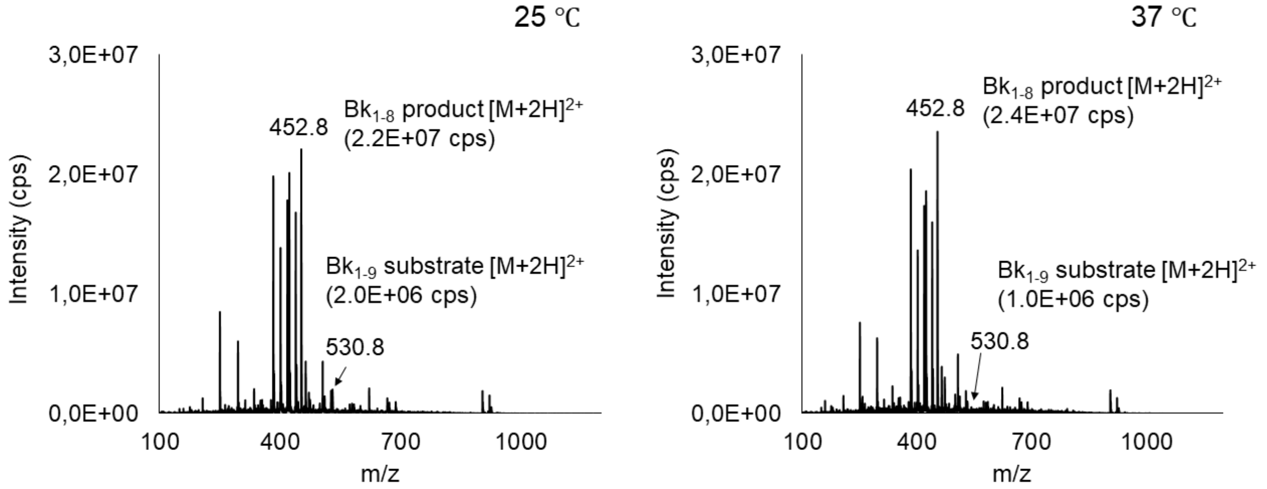

Fig. S5 ESI-MS analysis of the reaction solutions collected from CHT-IMERs operated at a flow rate of 5 μL/min at room temperature (25°C) and at physiological temperature (37°C). The substrate solution was 20 μM bradykinin in 20 mM ammonium acetate (pH 8.2)

References

[1] G.L. Ellman, Tissue sulfhydryl groups, Arch. Biochem. Biophys. 82 (1959) 70-77.
